# The Genotype-Tissue Expression (GTEx) Project: Linking Clinical Data with Molecular Analysis to Advance Personalized Medicine

**DOI:** 10.3390/jpm5010022

**Published:** 2015-02-05

**Authors:** Judy C. Keen, Helen M. Moore

**Affiliations:** Biorepositories and Biospecimen Research Branch, Division of Cancer Treatment and Diagnosis, National Cancer Institute, Bethesda, MD 20892, USA; E-Mail: moorehe@mail.nih.gov

**Keywords:** biobanking, biorepository, tissue biospecimen, gene variation, genotype, Genotype-Tissue Expression

## Abstract

Evaluation of how genetic mutations or variability can directly affect phenotypic outcomes, the development of disease, or determination of a tailored treatment protocol is fundamental to advancing personalized medicine. To understand how a genotype affects gene expression and specific phenotypic traits, as well as the correlative and causative associations between such, the Genotype-Tissue Expression (GTEx) Project was initiated The GTEx collection of biospecimens and associated clinical data links extensive clinical data with genotype and gene expression data to provide a wealth of data and resources to study the underlying genetics of normal physiology. These data will help inform personalized medicine through the identification of normal variation that does not contribute to disease. Additionally, these data can lead to insights into how gene variation affects pharmacodynamics and individualized responses to therapy.

## 1. Introduction

Personalized medicine is dependent on the link between genotypic and phenotypic data from an individual patient [1]. The central challenge to personalized medicine is to understand how genetic mutations and variability can directly affect phenotypic outcomes, the development of disease, or the determination of a tailored treatment protocol. In order to understand how a genotype affects gene expression and specific phenotypic traits, as well as the correlative and causative associations between such, data from a large number of participants must be collected and analyzed.

Two key questions must be addressed in the effort to develop treatment protocols that are tailored to an individual patient or subset of patients that carry the same trait. First, what is the impact of variability at both the genomic and gene expression levels in healthy and disease states? Second, how does genetic variability contribute to gene expression within individual tissues? To address these critical contributions to variability and normal physiology, the Genotype-Tissue Expression (GTEx) project was initiated in 2010 (http://commonfund.nih.gov/GTEx/index) [2].

Linkage of genetic variability to gene expression will correlate genetic and molecular research findings to specific clinical outcomes and identify the causative changes that lead to disease. These connections will help match patients with protocols in clinical trials, which can result in improved outcomes from disease.

GTEx is a Common Fund Project of the National Institutes of Health (NIH) that was designed to build the resources and infrastructure for researchers to address the question of how variability in genotype influences gene expression and phenotypic endpoints. The GTEx project has established a database of clinical and molecular information such as medical histories, expression data from RNA sequencing and whole exome sequences, and an associated tissue and cell line bank to store and distribute corresponding biospecimens. A list of the medical history, current medications, and infectious disease data collected for each donor can be found in Table 1.

This study is specifically and intentionally designed to understand the underlying genetic variability in predominantly healthy humans. Therefore, GTEx biospecimens and associated data can begin to address important questions surrounding how much variation exists in healthy tissues, what mutations are truly precursors to disease, or how and when a disease state arises. Understanding normal physiology will allow scientists to better understand disease pathology and lead to more accurate treatment for patients. To aid in dissemination and continued use of this data, all of the genetic, molecular, and clinical data from this study are publicly available through dbGaP (http://www.ncbi.nlm.nih.gov/gap), a database of genotypes and phenotypes managed by the National Library of Medicine. All data are deidentified, but linked through a public GTEx ID that is not traceable to the individual donor.

**Table 1:** List of clinical data associated with Genotype-Tissue Expression (GTEx) donor cases.

**Medical History**
Alzheimer’s OR Dementia
Ischemic Heart Disease (coronary artery disease (CAD), coronary heart disease, ischemic cardiomyopathy)
Cerebrovascular Disease (stroke, TIA, embolism, aneurysm, other circulatory disorder affecting the brain)
Heart attack, acute myocardial infarction, acute coronary syndrome
Renal Failure
Nephritis, Nephrotic Syndrome and/or Nephrosis
Chronic Respiratory Disease (Chronic Obstructive Pulmonary Syndrome (COPD) OR Chronic Lower Respiratory Disease (CLRD) (chronic bronchitis, emphysema))
Chronic Lower Respiratory Disease
Influenza (acute viral infection including avian influenza)
Pneumonia (acute respiratory infection affecting the lungs)
Diabetes mellitus type 1 (IDDM, formerly juvenile diabetes)
Diabetes mellitus type II (NIDDM, adult onset diabetes)
Uremia (Kidney Disorder)
Bacterial Infections (including septicemia (bacteria in the blood), meningococcal disease, staphylococcal infection, streptococcus, sepsis)
Liver Disease (liver abscess, failure, fatty liver syndrome, inherited liver insufficiency, acute/chronic hepatic insufficiency, necrobacillosis, rupture)
Arthritis
Major depression (unipolar depression, major depressive disorder)
Asthma
Hypertension
Parkinson’s
Schizophrenia
Crohn’s Disease
Gastric Reflux Disease, reflux esophagitis, heartburn, GERD
Atrial Fibrilation
Sjogren’s Disease (chronic dry mouth/dry eyes)
Diverticular Disease, diverticulitis
Ulcerative Colitis
Other data
**Current Medications**
Medication Name/Vitamins/Supplements
**Death Circumstances**
Is death certificate available
Date and time pronounced dead
Date and time of actual (witnessed) death
Date and time of presumed death
Date and time last seen alive
Place of Death
If death occurred outside of hospital, who determined date/time of death
Manner of Death
Death Classification: 4-point Hardy Scale
Did Coroner/ME Perform an Autopsy?
Was donor on a ventilator immediately prior to Death
If yes, specify duration (hours)
Death Certificate Cause of Death
Immediate Cause of Death
Approximate Interval: Onset to Death(hours)
First underlying Cause of Death
Approximate Interval: Onset to Death(hours)
Last Underlying Cause of Death
Was the body refrigerated at any time before procurement?
if yes, estimate number of hours in refrigeration (hours)
Organ Donor (OPO) Type
**Serology Report**
HIV I/II Ab
HIV I/II Plus O Antibody
HBsAg
HBsAb
HBcAb (Total; IgG+IgM)
HBcAb-IgM
HCV Ab
EBV IgG Ab
EBV IgM Ab
RPR
CMV Total Ab
HIV-1 NAT
HCV-1 NAT
PRR/VDRL

## 2. Specimen Collection, Clinical Data Elements, and Biospecimen Quality

This ambitious program has depended on a unique set of partnerships between organ procurement organizations, biorepository experts, pathologists, molecular biologists, geneticists, and statisticians. Details of the biospecimen and data collection have been previously reported [2]. Under the GTEx protocol, samples from 25–30 different tissues in the body are collected from recently deceased individuals. Each donor’s next-of-kin provides consent for the donation of tissues which includes an additional option to donate brain tissue. To date, tissues from over 650 unique donors, including over 250 brain samples, have been collected. The goal is to collect biospecimens from 900 unique donors. Clinical data are obtained from family members or medical records, if available. Data elements include general medical history, current medication status, death circumstances, and blood serology reports. Importantly, participants in this study are post-mortem donors unselected for any diseases, and most organs will be free of pathology. Making this clinical and molecular data available will provide valuable data for researchers to investigate how much variability exists in non-diseased tissues.

This study also provides tissue and cell line resources along with the clinical and molecular data to pursue pharmacodynamic and other studies that can contribute to personalized medicine. It has been long appreciated that genetic and environmental factors directly impact responses to therapy [3]. As sequencing has improved and more variants have been identified, the impact of gene mutation and gene expression variability in drug responses continues to be of critical importance to individualized treatment. While many challenges in establishing the impact of new variants and new information about gene expression on drug responses still exist, GTEx data and biospecimens can be used to shed light into identifying the critical elements that determine how an individual will react to a specific drug at a specific dose.

Biorepositories can link privacy protected clinical data from electronic health records to patient biospecimens that are stored in biorepositories to maximize the future use of the samples. Such linkage benefits research by allowing investigators to conduct studies that will correlate genetic and molecular research findings to specific clinical outcomes and identify the causative changes that lead to disease.

### Biospecimen Quality

Each tissue is preserved in PAXgene^®^ tissue fixative, embedded in paraffin, and histological sections are examined by a team of board certified pathologists for accuracy of tissue collection, determination of tissue quality, and evidence of disease. These images and histological data will be available to researchers in the future through the GTEx portal (www.gtexportal.org). PAXgene^®^ tissue-fixed samples are then shipped to the Laboratory Data Analysis Coordinating Center (LDACC) for whole genome and RNA sequencing. Following extensive quality control (QC), all deidentified genomic and clinical data is deposited into dbGaP for use by researchers worldwide. A manuscript providing detailed information about the quality and unexpected pathological findings observed in the GTEx samples is in preparation.

## 3. Anticipated Findings

Clinical data, biospecimens, and molecular analysis of GTEx samples are available to the research community through the GTEx portal and dbGaP (www.gtxportal.org and http://www.ncbi.nlm.nih.gov/gap). At this early juncture in the study, over 175 researchers have downloaded the available data from the dbGaP database and research grants have been awarded to investigators to conduct further genetic analyses. The first in-depth analysis of data is expected to be published in early 2015.

While many avenues of research can be pursued with this dense database, initial studies are focusing on the identification of tissue- and gender-specific expression Quantitative Trait Loci (eQTLs), on the degree of genomic variation that is observed in normal tissues, and on the level of gene expression variation in different tissue types. As the project matures and more data becomes available it will be possible to integrate clinical data, unanticipated findings, and molecular investigations to understand normal and disease states.

### 3.1. Expression Quantitative Trait Loci (eQTLs)

A major goal of the GTEx project is to identify and systematically analyze eQTLs in the human genome to understand the effects of variability on gene expression. Since eQTLs can directly link rare gene variants with gene expression, identifying eQTLs is more beneficial than the identification of gene mutations alone. In addition, understanding the regulation of gene expression and the role of regulatory elements in control of expression is essential for interpretation of genome wide association studies (GWAS). Together, GWAS, SNP and eQTL data can provide greater meaning to how gene variability controls gene expression and valuable insight into normal physiology and disease pathology to provide genomically informed decisions regarding personalized care and treatment for patients.

The GTEx Consortium has analyzed RNA sequencing data from over 1600 tissue samples from 175 individuals to date (GTEx consortium publication). From this analysis, thousands of tissue-specific and shared regulatory variants or eQTLs have been identified and characterized. This data provides a powerful tool to help define complex network relationships and to identify heterogeneous variation in and among human tissues. Since eQTLs and their association with gene expression can provide a link between genotype and phenotype, these data will enhance personalized medicine by providing crucial information that can lead to the identification of biomarkers, new therapies, or new diagnostic procedures.

### 3.2. Accessing the Samples: The GTEx Portal

While data generated from RNAseq and eQTL analysis is available for qualified investigators through dbGaP, additional information about the samples, limited donor characteristics, and eQTL data is available through the GTEx portal (www.gtexportal.org). This site allows researchers to search within or across tissues for eQTLs and expression levels. The portal also provides a searchable platform for obtaining information about biospecimens, DNA, and/or RNA availability to facilitate outside investigators’ utilization of these valuable resources. The GTEx Access Policy can be found at http://www.gtexportal.org/home/samplesPage.

## 4. Conclusions

This extensive collection of biospecimens and associated data from healthy individuals provides a tremendous resource for scientists. The GTEx study is truly a unique program. No other resource of this kind is currently available. With the cell lines, frozen biospecimens, PAXgene Tissue-fixed tissues, genotypic and gene expression data, and clinical data available for each donor, many scientific questions can be addressed. Not only does this study provide normal tissues that can be used as non-diseased controls in experiments to understand disease states, but this collection can be used to address human variability. How much genomic or gene expression variability exits in healthy humans? How much impact does gene mutation have on gene expression levels? Do genetic mutations manifest differently in different tissues? Is there a commonality of expression or genetic variation between phenotypically different tissues? What can genetic and gene expression levels tell us about disease? How can understanding healthy physiology, genomic, and molecular data inform treatment options or the development of new treatment protocols? When does disease arise? Are there new ways to control expression?

In addition to the clinical and genomic/gene expression data amassed, researchers are also investigating the ethical, legal, and social implications (ELSI) of biobanking and use of biospecimens in long-term, as-yet-to-be defined research studies. The ELSI studies are exploring what families, communities, and decision makers know or understand about biobanking, biological studies, and long-term storage of biospecimens. The ELSI team is also examining the consent process and how well family decision makers remember the information that is provided to them. As genomic and molecular data become more prevalent in clinical medicine and more accessible to patients, understanding what the public knows and how they feel about contributing to research will be invaluable to improve the consent and overall research process.

GTEx aims to finish collection of biospecimens by mid-late 2015 and have all of the molecular analysis complete by late 2016. This, however, is just the beginning for this data set. Many researchers will access the data and use the biospecimens to conduct a number of studies and to advance patient care by providing a comprehensive understanding of human health and disease at the individual level.

After collections are completed, the GTEx project will continue to be an important resource for investigators. Additional projects have already been awarded to use the biospecimens and data that has been generated. This allows for further characterization of the samples via use of other ‘omics analysis such as epigenetics, proteomics, and metabolomics characterization. Biospecimens, including cell lines, frozen tissues, and PAXgene Tissue preserved tissues can be obtained through the GTEx access policy. The use of these biospecimens, especially when combined with data in dbGaP will increase the overall understanding of human biology. It will advance personalized medicine by providing insight into the context of disease biomarkers in normal physiology by understanding how disease may develop, and how to use biomarkers to detect or treat disease. As the data continues to be compiled and used, the benefit to research and science will only be enhanced. Creating a resource that allows for the use of many platforms, from genotyping to methylation and proteomics, is powerful. Along with a rich set of clinical data, this project will provide a growing resource of data to enhance personalized medicine.
